# Actin-binding protein regulation by microRNAs as a novel microbial strategy to modulate phagocytosis by host cells: the case of N-Wasp and miR-142-3p

**DOI:** 10.3389/fcimb.2013.00019

**Published:** 2013-06-05

**Authors:** Paulo Bettencourt, Sabrina Marion, David Pires, Leonor F. Santos, Claire Lastrucci, Nuno Carmo, Jonathon Blake, Vladimir Benes, Gareth Griffiths, Olivier Neyrolles, Geanncarlo Lugo-Villarino, Elsa Anes

**Affiliations:** ^1^Centro de Patogénese Molecular, Faculdade de Farmácia, Unidade dos Retrovírus e Infecções Associadas e Instituto de Medicina Molecular, Universidade de LisboaLisboa, Portugal; ^2^Institut Cochin, Centre National de la Recherche Scientifique, Institut National de la Santé et de la Recherche Médicale U 1016, UMR 8104, Université Paris DescartesParis, France; ^3^Centre National de la Recherche Scientifique, Institut de Pharmacologie et de Biologie StructuraleToulouse, France; ^4^Institut de Pharmacologie et de Biologie Structurale, Université de Toulouse, Université Paul SabatierToulouse, France; ^5^Gene Core Facility, European Molecular Biology LaboratoryHeidelberg, Germany; ^6^Department of Molecular Biosciences, Blindern, University of OsloOslo, Norway

**Keywords:** phagocytosis, N-Wasp, miRNA, miR-142-3p, tuberculosis, macrophage, *M. tuberculosis*, *M. smegmatis*

## Abstract

*Mycobacterium tuberculosis* (Mtb) is a successful intracellular pathogen that thrives in macrophages (Mφs). There is a need to better understand how Mtb alters cellular processes like phagolysosome biogenesis, a classical determinant of its pathogenesis. A central feature of this bacteria's strategy is the manipulation of Mφ actin. Here, we examined the role of microRNAs (miRNAs) as a potential mechanism in the regulation of actin-mediated events leading to phagocytosis in the context of mycobacteria infection. Given that non-virulent *Mycobacterium smegmatis* also controls actin filament assembly to prolong its intracellular survival inside host cells, we performed a global transcriptomic analysis to assess the modulation of miRNAs upon *M. smegmatis* infection of the murine Mφ cell line, J774A.1. This approach identified miR-142-3p as a key candidate to be involved in the regulation of actin dynamics required in phagocytosis. We unequivocally demonstrate that miR-142-3p targets N-Wasp, an actin-binding protein required during microbial challenge. A gain-of-function approach for miR-142-3p revealed a down-regulation of N-Wasp expression accompanied by a decrease of mycobacteria intake, while a loss-of-function approach yielded the reciprocal increase of the phagocytosis process. Equally important, we show Mtb induces the early expression of miR-142-3p and partially down-regulates N-Wasp protein levels in both the murine J774A.1 cell line and primary human Mφs. As proof of principle, the partial siRNA-mediated knock down of N-Wasp resulted in a decrease of Mtb intake by human Mφs, reflected in lower levels of colony-forming units (CFU) counts over time. We therefore propose the modulation of miRNAs as a novel strategy in mycobacterial infection to control factors involved in actin filament assembly and other early events of phagolysosome biogenesis.

## Introduction

In the arms race of host-microbe coevolution, pathogens such as *Mycobacterium tuberculosis* (Mtb) have evolved ingenious strategies to survive inside the host. Prominent among these strategies is the subversion of macrophages (Mφs), which play a dual role as the primary host cell for microbial replication and as the crucial effector cell in the immune response against this obligate intracellular pathogen. Mφs are ideal targets for subversion since they are endowed with bacterial killing mechanisms, such as the exposure of the invading microbes to a hostile intracellular environment, such as occurs following fusion of phagosomes with acidic and hydrolase-rich lysosomes (phagolysosomes). Perhaps the best-known strategy for any invading bacterial pathogen is to manipulate the early steps of the interaction with Mφs, in order to avoid the activation of the microbiocidal mechanisms. This is indeed the case for Mtb as the inhibition of phagolysosome biogenesis in infected Mφs is a classical pathogenesis determinant (Deretic et al., 2004).

We, and others, have shown that in phagolysosome biogenesis there are at least three distinct processes inhibited by mycobacteria: phagosomal actin assembly, fusion with lysosomes, and acidification (Sturgill-Koszycki et al., 1994; Anes et al., 2003, 2006; Castandet et al., 2005; Kozomara and Griffiths-Jones, 2011). A central feature to this pathogenic strategy is the manipulation of actin's fate within Mφs such that it favors bacterial survival. In Mφs, actin filament assembly is required for pseudopodia provided evidence that phagosomal membranes provide tracks for lysosomes to move toward the actin nucleating organelle (Anes et al., 2003; Kjeken et al., 2004). In addition, actin filament assembly also plays a role in the pro-inflammatory response. Several signaling lipids, cAMP, extracellular ATP and the P2X7 receptor, were shown to be involved in actin assembly and the killing/survival of pathogenic mycobacteria (Kalamidas et al., 2006; Treede et al., 2007; Jordao et al., 2008a,b; Kühnel et al., 2008; Kuehnel et al., 2009). Furthermore, some lipid effectors that regulate actin assembly also control NF-κB, a transcription factor involved in the pro-inflammatory response (Gutierrez et al., 2009). While these observations suggest a central role of actin assembly in boosting the ability of Mφs to kill mycobacteria, the mechanism(s) of how Mtb or Mφs control actin-mediated dynamic events during infection remain relatively unknown.

Considering the temporal aspects of the phagocytic process it must be appreciated that many of the processes occur at quite different time scales. A single round of phagocytosis occurs much faster (in a few minutes) than a transcriptional response resulting in protein synthesis (at least 10 min, and up to many hours). The earliest measurable event in phagocytosis is actin assembly, a highly sophisticated process that generally occurs on the surface of cellular membranes; it occurs at the time scale of seconds. Therefore, the mechanisms influencing these early events of the microbe-Mφ interaction must be equally dynamic in terms of time and efficiency. Recently, a new class of regulators has emerged as key participants in controlling cellular processes: the microRNAs (miRNAs). These are small non-coding, single-stranded RNAs (around 22 nucleotides length), which act by specifically binding to the 3′-UTR regions of target mRNAs, causing translational repression or mRNA degradation, along with subsequent reduction in protein expression and thereby function. The miRNAs are emerging as important subject of investigation due to their roles in development, cancer (Williams, 2008), metabolic and neurologic disorders (Boissonneault et al., 2009), cardiac regeneration (Eulalio et al., 2012a). They are also accepted as playing key roles in inflammatory responses (O'Connell et al., 2012); among other biological activities.

Especially relevant to the present study are the observations implicating miRNAs in the regulation of the mRNA levels of actin-binding proteins (ABP) and other factors involved in actin-mediated events. For instance, miR-21 targets the mRNA for the tropomyosin (Zhu et al., 2007); both miRNA-143 and miR-145 regulate podosome formation in smooth muscle cells (Xin et al., 2009); and miR-145, miR-133a, and miR133b target the fascin homolog 1 (Kano et al., 2010). Moreover, cofilin is indirectly regulated by miR-205 via Rho-ROCKI activity in keratinocytes (Yu et al., 2010). Other known examples include that of MiR-132 regulating Rac1 activity and hippocampal spine formation (Impey et al., 2010), and miR-206 targeting the mRNA for the GTPase, Cdc42, in a breast cancer cell line (Liu et al., 2010). Finally, the WASP family member WAVE3, an actin cytoskeleton remodeling and metastasis promoter protein, is regulated by miR-200 (Sossey-Alaoui et al., 2009). Therefore, it is plausible that microbial pathogens might manipulate actin-dependent events through the modulation of the host miRNAs to enhance their survival within Mφs.

In the context of host-pathogen interactions, the pathogenic capacity to alter the host miRNA repertoire is most clearly seen in the considerable progress done in the context of viral and parasitic infections (Cullen, 2011; Hakimi and Cannella, 2011). Yet, in comparison to these two types of microbial infections, the host miRNA response to bacterial pathogens has been less explored (Eulalio et al., 2012b). Perhaps the importance of miRNAs in the host response against bacterial infections is best illustrated in the case of *Salmonella*. Indeed, this intracellular bacterium triggers specific alterations in the miRNA repertoire in Mφs, such as the down-regulation of the Let-7 miRNA gene family members that serve as a post-transcriptional brake to IL-6 and IL-10 secretion, thereby modulating the immune response in favor of the pathogen (Voinnet, 2011; Eulalio et al., 2012b). Recently, however, there has been also an increase interest in the role of miRNA in mycobacterial infections. This includes the identification of miRNAs as biomarkers for tuberculosis (TB) at different stages (Fu et al., 2011; Wang et al., 2011; Qi et al., 2012; Yi et al., 2012; Spinelli et al., 2013), and the modulation of the miRNA repertoire during host cell infection with Mtb (Rajaram et al., 2011; Singh et al., 2013), Bacillus Calmette-Guerin (BCG) (Ma et al., 2011; Wu et al., 2012) and *M. avium* (Sharbati et al., 2011). In particular, the regulatory effect on pro-inflammatory cytokine production via the mIR-125b/mIR-155 axis represents the best described strategy of how Mtb subverts host immunity and potentially enhances its virulence. On the one hand, Mtb blocks the biosynthesis of TNFα and prevent its pro-inflammatory consequences by increasing inducing high levels of mIR-125b through lipomannan (the major cell wall component) secretion. On the other hand, the lipomannan from the non-pathogenic *M. smegmatis* fails to affect the mIR-125b expression, and instead, the host miRNA response is characterized by the induction the mIR-155 expression, which enhances TNFα mRNA half-life and translation, resulting in a stronger microbiocidal outcome (Rajaram et al., 2011).

To our knowledge, there are no reports in the literature concerning the role of miRNAs in the mRNA regulation of ABPs associated with early events in bacterial phagocytosis leading to phagolysosome biogenesis. Based on previous work with *M. smegmatis* as a model of phagocytosis, we found out that this non-virulent mycobacterial strain is able to modulate the actin cytoskeleton and the pro-inflammatory response in such as way as to enable its intracellular survival up to 2 days, despite the fact that it cannot prevent its ultimate elimination from the host cell (Anes et al., 2003, 2006; Jordao et al., 2008a). The ability of the host cell to assemble actin from the membrane of the phagosome is directly related with additional fusion events with lysosomes that lead to the killing of mycobacteria. We therefore decided to investigate the role of miRNAs as a potential novel mechanism in the regulation of actin-mediated events influencing the process of phagocytosis within the context of mycobacteria infection, including both non-virulent and virulent species.

## Materials and methods

### Cell preparation, cell lines and bacterial culture conditions

The mouse Mφ cell line J774A.1 was cultured as described previously (Anes et al., 2003). The human monocyte derived Mφs were obtained from healthy blood donors (Instituto Português do Sangue, Lisbon, Portugal), and differentiated following a previously published procedure (Wang et al., 2010). A protocol of collaboration was established between Drs. Anes and Castro (the head of the Portuguese Institute for Blood in 2007), in order to have access to buffy coats from blood donors for scientific research. Alternatively, monocytes were obtained from healthy blood donors Etablissement Français du Sang (EFS) in Toulouse, France. Written informed consents were obtained from the donors under EFS contract n°21/PVNT/TOU/IPBS01/2009-0052. Following articles L1243-4 and R1243-61 of the French Public Health Code, the contract was approved by the French Ministry of Science and Technology (agreement nuAC 2009-921). The monocytes were differentiated into Mφs following a previously published protocol (Tailleux et al., 2003).

The strain *Mycobacterium smegmatis* mc^2^155, containing a p19 (long lived) EGFP plasmid was kindly provided by Dr. Douglas Young (London School of Hygiene and Tropical Medicine, London, UK), and the green fluorescent protein (GFP)-expressing strain of *M. tuberculosis* (H37Rv-pEGFP) plasmid was a kind gift from G. R. Stewart (University of Surrey, United Kingdom). *M. smegmatis* was grown in medium containing Middlebrook's 7H9 broth Medium (Difco, USA), Nutrient broth (Difco, USA) supplemented with 0.5% glucose and 0.05% Tween 80 at 37°C on a shaker at 200 r.p.m. (Anes et al., 2003). Bacteria were sub-cultured every day in fresh medium before use. *M. tuberculosis* H37Rv was grown in Middlebrook's 7H9 medium (Difco) and supplemented with 10% OADC Enrichment (Oleic acid; Albumin Factor V, Bovine; Dextrose; Catalase Powder; Sodium Chloride) (Difco, USA) (Anes et al., 2003).

### Mφ infection

Bacterial cultures in exponential growth phase were spin-down, washed in phosphatebuffered saline (PBS, without Ca2+ or Mg2+, GIBCO Invitrogen) and resuspended in Dulbecco's Modified Eagle Medium (DMEM). Bacterial clumps were removed by incubation in an ultrasonic water bath for 15 min, followed by a low speed centrifugation for 2 min. Mφs were seeded onto 24-well tissue culture plates, 5 × 10^5^ cells/well, for protein and RNA extraction. For immunofluorescence (IF), 0.5 × 10^5^ cells/well were seeded into cover slips and incubated for overnight until reach 1.5 × 10^5^ cells per cover slip. Mφs were infected with a single-cell suspension of mycobacteria at multiplicity of infection (MOI) of 10:1 (10 bacteria per Mφ). Bacteria were internalized by Mφs during 1 h (infection experiment), at 37°C with 5% CO_2_. In every experiment, after 1 h of infection, cells were washed with PBS and maintained in DMEM with Gentamycin (10 μg/ml) to kill extracellular bacteria.

The colony-forming units (CFU) assay in Mφs was performed following a previously published proceduture (Botella et al., 2011). Briefly, Mφs were incubated with bacteria in RPMI-10% AB serum (MOI 0.1 for human cells and 10 for mouse cells) and for various times (see text). For bacterial proliferation experiments, after a 4-h infection with H37Rv-eGFP, Mφs were washed with PBS to remove extracellular bacteria, and were then incubated in fresh medium. At the indicated time points, cells were lysed with 500 μ l of 0.1% Triton X-100 in sterile water, and viable intracellular bacteria were counted by plating serial dilutions of the lysates onto Middlebrook 7H11 agar-10% OADC.

Alternatively, the rate of phagocytosis of H37Rv-pEGFP by human Mφs was measured after 4 h of infection (MOI 10) by flow cytometry using a Becton Dickinson LSRII flow cytometer using the FlowJo software.

### MicroRNA expression

Total RNA was isolated using TRIzol reagent (Invitrogen, Paisley, Scotland) according to the manufacturer's instructions. RNA quality was controlled using the RNA 6000 Pico LabChip kit (Agilent, Waldbronn, Germany) and quantified with a NanoDrop ND-1000 Spectrophotometer (Nanodrop Technologies, Wilmington, DE, USA). Microarray results were submitted to GEO repository with the GEO accession number GSE23429.

### RT-qPCR

Messenger RNA relative quantification started with 1 μg of total RNA that was used for random hexamer primed cDNA synthesis (SuperscriptTM II reverse transcriptase, Invitrogen) according to the manufacturer protocol. Amplification was detected using SYBR Green PCR master mix (Applied Biosystems) and different sets of primers (MWG) at a final concentration of 0.5 μM. The PCR settings used: 1 cycle of 95°C for 10 min, followed by 40 cycles of 95°C for 15 s, 60°C for 30 s, and 72°C for 30 s. The mRNA expression profiles were normalized with respect to GAPDH (Glyceraldehyde 3-phosphate dehydrogenase). The qPCR was performed using an ABI 7500 Real Time PCR System (Applied Biosystems) and data was collected at the amplification step and analysed with SDS v1.2. Software. Fold increase of each gene was calculated using the −2^−ΔΔCt^ method.

The specific relattive quantification of miR-142-3p in total RNA samples was based on TaqMan MicroRNA Assays from Applied Biosystems (ABI) in our laboratory or by final report analysis provided by EXIQON (DK) microRNA qPCR services. Briefly, 10 ng of total RNA were used for cDNA synthesis, according to the manufacturer protocol. The qPCR was run with the following steps: 1 cycle of 95°C for 10 min, followed by 40 cycles of 95°C for 15 s, and 60°C for 15 s, without dissociation stage. The miRNA expression profiles were normalized either to reference gene U6 (snRNA) or to the average obtained between miR-23a, miR-23b, and miR-24, whose expression levels are stable under the experimental conditions applied in this study. The mean and standard deviation over all the median normalized intensity data obtained from the microarray was calculated. The data was filtered so that the mean expression of the median normalized intensity value is high (higher than 10), and that the standard deviation is low (15 % of the mean).

### Identification of the specificity of miR-142-3p to the 3′-UTR mRNA of N-WASP

A 356 bp fragment of 3′-UTR of Wasl mRNA, containing the seed sequence (ACACTAC) of miR-142-3p was amplified by PCR (forward primer 5′-GCGACGTCGGTGAAATACTAAACACTACTTC-3′, reverse primer 5′-CCCTCGAGGTACAGAAAAAGTAGGGTATG-3′). The fragment was designated as Wasl 3′-UTR and inserted into the pmirGLO dual-luciferase miRNA target expression vector (Promega), between the *SacI* and *Xho*I restriction sites. Mutant plasmids were constructed bearing a single point mutation on the primer that includes the seed sequence, thus incorporating this mutation on the amplicon during the PCR amplification. The forward primer 5′-GCGAGCTCGGTGAAATACTAAACATTACTTC-3′ for Wasl 3′-UTR plasmid was used. Point mutation is underlined.

### Transient transfection

The miR-142-3p mimics and inhibitors (Dharmacon, Lafayette, CO, USA) were used for transient transfection in gain or loss-of-function experiments, respectively. The negative control sequence (scramble), was designed by the supplier, and was confirmed to have minimal sequence identity with miRNAs in human, mouse and rat. Either 100 nM of synthesized oligonucleotide or 10 ng/μl of plasmid were mixed with 1.5 μl of Dharmafect4 (Dharmacon, Lafayette, CO, USA) per 400 μl of serum free DMEM and transfected into 1.5 × 10^5^ cells. The transfection efficiency achieved was approximately 90%, as evaluated by confocal microscopy using a miR-142-3p inhibitor labeled with Alexa 594 fluorochrome (Exiqon, Vedbaek, Denmark). After transfection, the cells were allowed to recover by incubating either for 48 or 72 h at 37°C, for total RNA of phenotypic assays.

For the Dual-Luciferase assay, the constructed plasmids were co-transfected, with the mimics for miR-142-3p or scramble (Dharmacon, Lafayette, CO, USA), into HEK293t cells. The reporter assay was performed using the Dual-Luciferase Reporter Assay System (Promega), according to manufacturer's instructions.

### Immunofluorescence

Cells were fixed with 4% paraformaldehyde, 4% sucrose solution in PBS for 30 min, and quenched by incubating with PBS 50 mM NH_4_Cl. Then cells were permeabilized with 0.1% Triton in PBS for 5 min. Fixed cells were washed and blocked with 1% BSA in PBS and incubated with rhodamine-phalloidin for F-actin staining and 4′,6-diamidino-2-phenylindole, dihydrochloride (DAPI) for nuclear staining (both from Molecular Probes, Invitrogen, UK), in 1% BSA/PBS for 30 min. Cells were mounted with Dako mounting media and analysed by confocal microscopy (Zeiss LSM510 META).

### Western blot

Cells plated in 24-well plates, under different conditions, were washed twice with PBS, and harvested in 250 μl of ice-cold, non-denaturating lysis buffer (TRIS 50 mM, NaCl 150 mM, Triton 1%, EDTA 1 mM, Protease Inhibitor Cocktail Tablets from Roche, Mannheim, Germany). Lysates were collected after 30 min of incubation and spun down for 5 min at 12000 × g, to remove debris of broken cells. The supernatant was collected and the protein concentration was measured using Bradford method. Approximately 20 μg of protein extracts were subjected to electrophoresis in 10% SDS-PAGE gels, transferred to a nitrocellulose membrane and blocked with 0.1% Tween20, 5% of low fat milk Tris Buffered Saline (TBS). The nitrocellulose membrane was then incubated with the primary antibodies, anti- N-Wasp, Cdc42, or Tubulin rabbit monoclonal antibodies (Cell Signaling, USA). For the confirmation of siRNA-mediated inactivation of N-Wasp, total protein lysates were extracted in the same manner as above. Proteins were separated with 4–12% Bis-Tris Gel (Invitrogen), transferred onto nitrocellulose membranes and incubated with anti-N-WASP (H100, Santa Cruz Biotechnology, 1/200), anti-actin (A5060, Sigma, 1/10000) overnight at 4°C. All membranes were washed and incubated with secondary HRP-conjugated antibodies. The bands were visualized with a chemiluminiscence reagent (Amersham Biosciences, UK) and quantified using Adobe Photoshop CS3 software.

### SiRNA-mediated gene silencing

Human Mφs were transfected with the siRNA (final concentration of 133 nM) using the Hiperfect transfection reagent according to the protocol we have developed and optimized (Lefèvre et al., 2013). This siRNA targeted the following human genes (all SMARTpool from Dharmacon): WASL and a non-targeting/scramble. This protocol resulted in a transfection efficiency of nearly 100% and a survival rate ranging no less than 85%, as determined by flow cytometry of cell transfected with siGLO RISC-free siRNA (Dharmacon) and the Anexin-V kit (Miltenyi Biotec). Upon 6 h of transfection with these siRNAs, the reaction was stop by adding medium with the presence of MCSF (10 ng/ml) (Miltenyi Biotec). After 96 h, MDMs were used for experiments. The gene silencing effect lasted up to 7 days with no significant toxicity to MDMs. Functional gene silencing was verified by western blot analysis as described above.

### Analysis of microarray data

GPR files produced from GenePix Prov V 6.0 software (Molecular Devices) analysis of scanned tif images were parsed, combined and the data median normalized using the MiChip library of BioConductor (http://www.bioconductor.org/help/bioc-views/release/bioc/html/MiChip.html). The median of the 3 uninfected replicates was taken and used to produce log2 ratios with respect to the normalized data from infected cells. The log2 ratio data was then analysed using Significance Analysis of Microarrays (SAM) (Tusher et al., 2001) with a False Discovery Rate (FDR) < 1% using the TigrTools MeV package (Saeed et al., 2006) and hierarchal clustered using the same application.

### Statistical analysis

Data are presented as mean either ± SD or ± SEM of at least three independent experiments; *p*-values (Student's *T*-Test) are relative to the control. Statistical significance was assumed when *P* < 0.05.

## Results

### Expression of miR-142-3p is induced in murine Mφs upon mycobacteria infection

**Figure 1:**
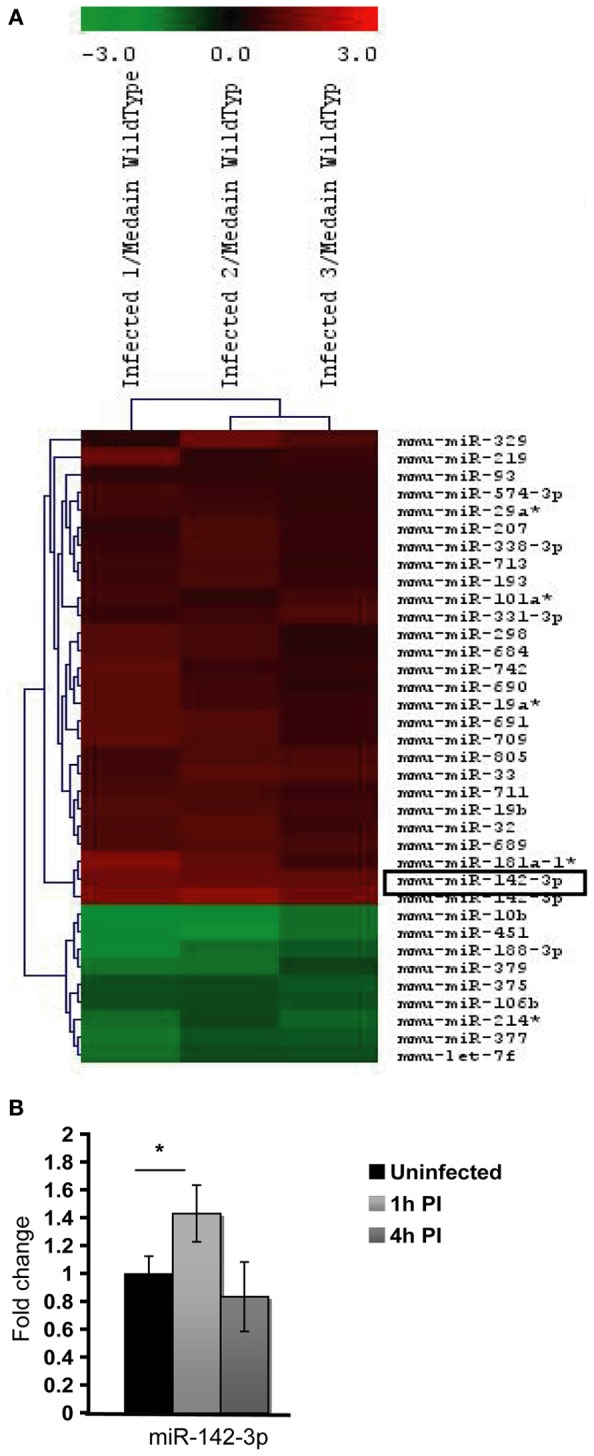
**MicroRNA Expression in J774A.1 Macrophages infected with *M. smegmatis* for 1 h. (A)** Heatmap of the most significantly regulated genes. The median normalized intensity values for each of the three infected replicates were divided by the median of the uninfected (wild type) samples. The ratio was then converted to log2 space and changes in the expression ratio were analysed using the Significance Analysis of Microarrays Test to isolate those with significant changes, FDR < 1%. **(B)** Relative expression of miR-142-3p in mouse cells infected with *M. smegmatis* at MOI 10, as measured by qPCR analysis. Data is represented as the mean fold change per sample ± SD at 1 and 4 h post-infection (^*^P ≤ 0.05).

To discern whether miRNAs are involved in phagocytosis during the context of mycobacterial infection, we first decided to assess the modulation of miRNAs via a global transcriptomic analysis. Previously, we demonstrated that the non-virulent *M. smegmatis* is able to modulate actin filament assembly in order to prolong its intracellular survival up 2 days before it succumbs to the diverse antimicrobial strategies employed by Mφs to eliminate intracellular pathogens (Anes et al., 2003, 2006; Jordao et al., 2008a). Since virulent Mtb is well known to block actin filament assembly to facilitate its survival in the host cell, we reasoned that the control mechanisms of actin filament assembly might be a shared feature among these mycobacterial strains. For this reason, and the fact that *M. smegmatis* is an easier model to work with (i.e., a fast doubling time and a biosafety level 1 laboratory requirement), we performed microarrays analysis. For this we infected the murine Mφ cell line J774A.1 with this non-virulent bacterium for 1 h. As shown in Figure 1A and Table 1, this approach revealed the modulation of 36 miRNAs, of which 27 were induced and 9 down-regulated.

**Table 1:** **MicroRNA fold change regulation**.

**Name**	**Regulation (fold change)**	
	**Positive**	**Negative**
mmu-miR-19b	1,67075	ND
mmu-miR-805	1,64086	ND
mmu-miR-142-3p	2,37443	ND
mmu-miR-181a-1^*^	1,9049	ND
mmu-miR-742	1,87794	ND
mmu-miR-298	1,7619	ND
mmu-miR-193	1,28083	ND
mmu-miR-101a^*^	1,64493	ND
mmu-miR-207	1,72828	ND
mmu-miR-329	1,77354	ND
mmu-miR-219	2,24679	ND
mmu-miR-19a^*^	1,79323	ND
mmu-miR-684	1,74488	ND
mmu-miR-29a^*^	1,4705	ND
mmu-miR-32	1,94128	ND
mmu-miR-33	1,99592	ND
mmu-miR-574-3p	1,5345	ND
mmu-miR-689	1,84419	ND
mmu-miR-331-3p	1,70916	ND
mmu-miR-690	1,86768	ND
mmu-miR-691	1,9171	ND
mmu-miR-93	1,42542	ND
mmu-miR-142-5p	2,51666	ND
mmu-miR-709	1,89836	ND
mmu-miR-713	1,77604	ND
mmu-miR-338-3p	1,59173	ND
mmu-miR-711	1,72723	ND
mmu-miR-106b	ND	1,88146
mmu-miR-379	ND	2,45017
mmu-let-7f	ND	1,97928
mmu-miR-375	ND	1,75779
mmu-miR-377	ND	2,21627
mmu-miR-10b	ND	2,36679
mmu-miR-451	ND	2,5183
mmu-miR-188-3p	ND	2,12642
mmu-miR-214^*^	ND	1,84229

ND, Not detected.

The fold change is calculated by the ratio between the average of the microarray data of the infected cells, and the average of the microarray data of the non-infected cells. The differential expression for each gene is statistically significant (P ≤ 0.05).

Next, in order to select the key potential miRNA candidates involved in the regulating actin dynamics required for phagocytosis, we applied the following criteria: (1) the quality of the raw data was considered by looking for midrange expression in order to avoid the effects of low level expression changes due to background or high level saturation; (2) to ensure reproducibility we used data based on triplicates with acceptable low standard deviations; (3) the miRNAs should be highly conserved across species; and the mRNA targets should be both (4) relevant early on during bacteria internalization and (5) encode for ABPs. Based on these criteria, the miR-142-3p was selected as the best candidate for further study. Not only was its level of expression level acceptable (~2.4 fold change) but also it represented one of the highest modulated miRNA's in our microarray analysis (Figure 1, Table 1); it was also predicted to bind ABPs involved in phagocytosis, as explained below. Moreover, miR-142-3p expression is associated to normal myeloid leukocyte differentiation (Careccia et al., 2009), and among its *bona fide* gene targets in the immune system, include the pro-inflammatory cytokine IL-6 that plays an essential role in protective and pathological immune responses (Sun et al., 2011). To further validate the induction of this miRNA, we performed qPCR analysis in J774A.1 Mφs infected with *M. smegmatis* at different time points and compared their expression levels to uninfected cells. As shown in Figure 1B, we observed at 1 hpi a slight, but significant induction miR-142-3p expression that was, however, short-lived: it returned to basal levels at 4 hpi. Altogether, while the expression levels for this miRNA in J774A.1 Mφs infected with *M. smegmatis* was slightly lower in the qPCR analysis compared to those obtained from the microarray data, they supported the notion that miR-142-3p is up-regulated at the earliest stage of infection.

The miR-142-3p is predicted to target two mRNAs encoding for ABPs, cofilin2 (Cfl2) and Wiskott-Aldrich Syndrome-Like (human) (N-Wasp), which are involved during the early events of phagocytosis (McGee et al., 2001; Caron et al., 2006; Park and Cox, 2009; Dart et al., 2012). These two targets were consistently present in all in 5 miRNA target prediction databases used in this study: Targetscan (Lewis et al., 2005), miRDB (Wang, 2008; Wang and El Naqa, 2008) and microRNA.org (Betel et al., 2008), Diana (Maragkakis et al., 2009a,b) and PicTar (Krek et al., 2005). Furthermore, additional predicted targets from all 5 databases were combined and the intersections computed. Out of 2548 distinct genes, only 11 overlapped in all 5 prediction sets (Table 2). The combined list of distinct gene targets (2548 genes, 2275 mapped to GO terms) derived from the 5 miRNA target databases was further analysed for enrichment of molecular function Gene Ontology terms using the Genomatix Genome Analyser GeneRanker tool (www.genomatix.de) El Dorado version 1210. GO terms selected have a probability of enrichment less than 0.01. This analysis showed that ABPs and cytoskeletal-binding proteins are indeed strongly enriched as potential targets of the miR-142-3p (Table 3).

**Table 2:** **The detailed listing of the 11 genes that appear in the 5 databases**.

**Gene**	**Name**	**Accession number**	**GO function**	**DB counts**
TMEM59	Transmembrane protein 59	NM_029565	Molecular function	5
ASH1L	Ash1 (absent, small, or homeotic)-like (Drosophila)	NM_138679	DNA-binding, histone-lysine N-methyltransferase activity, metal ion-binding, methyltransferase activity, molecular function, transferase activity, zinc ion-binding	5
STRN3	Striatin, calmodulin-binding protein 3	NM_052973, AK140447	Armadillo repeat domain-binding, calmodulin-binding, protein complex-binding, protein phosphatase 2A-binding, sequence-specific DNA-binding transcription factor activity, transcription repressor activity	5
**CFL2**	**Cofilin 2, muscle**	**NM_007688**	**Actin-binding, molecular function**	**5**
RGL2	Ral guanine nucleotide dissociation stimulator-like 2	NM_009059	Guanyl-nucleotide exchange factor activity	5
LRRC1	Leucine rich repeat containing 1	NM_172528, BC046591	Molecular function	5
**WASL**	**Wiskott–Aldrich syndrome-like (human)**	**NM_028459**	**Actin-binding, protein-binding, small GTPase regulator activity**	**5**
EHF	Ets homologous factor	NM_007914, AF035527, BC005520	DNA-binding, sequence-specific DNA-binding, sequence-specific DNA-binding transcription factor activity	5
SH3GLB1	SH3-domain GRB2-like B1 (endophilin)	NM_019464, AF272946	SH3 domain-binding, cytoskeletal adaptor activity, fatty acid-binding, lipid-binding, lysophosphatidic acid acyltransferase activity, protein-binding, protein homodimerization activity	5
CPEB2	Cytoplasmic polyadenylation element-binding protein 2	NM_175937, AK042065	RNA-binding, nucleic acid-binding, nucleotide-binding, poly-pyrimidine tract-binding	5
INPP5A	Inositol polyphosphate-5-phosphatase A	NM_183144	PH domain-binding, inositol-polyphosphate 5-phosphatase activity	5

List of genes predicted as targets for mmu-miR-142-3p were downloaded from the five miRNA target data bases (Diana, Targetscan, PicTar, micrornaorg and mirDB). Bold entries represent actin binding proteins potential targets of miR-142-3p

**Table 3:** **Molecular functions go term enrichment analysis of predicted gene targets of mmu-miR-142-3p**.

**GO-term**	**GO-term ID**	***P*-value**	**Adjusted *p*-value**	**No. of genes (observed)**	**No. of genes (expected)**	**No. of genes (total)**	**List of observed genes**	**Gene IDs**
Binding	GO:0005488	1,62E-08	n/a	34	15,55656796	10244	Slc1a3, Strn3, Cfl2, Sh3glb1, Aff2, Wasl, Bach2, Stx12, Myh10, Mllt1, Atg16l1, Gnb2, Clcn5, Cyb5r3, Rab3a, Cask, Ash1l, Cpeb2, Atf7ip, Tsen34, Tfg, Tgfbr1, Prlr, Tardbp, Ehf, Mgat4a, Ppp1r10, Lrrc1, Mark3, Marcks, Pde4b, Crk, Arntl, Fntb	20512, 94186, 12632, 54673, 14266, 73178, 12014, 100226, 77579, 64144, 77040, 14693, 12728, 109754, 19339, 12361, 192195, 231207, 54343, 66078, 21787, 21812, 19116, 230908, 13661, 269181, 52040, 214345, 17169, 17118, 18578, 12928, 11865, 110606
Protein-binding	GO:0005515	9,19E-08	n/a	24	8,148823083	5366	Slc1a3, Strn3, Cfl2, Sh3glb1, Wasl, Bach2, Stx12, Myh10, Mllt1, Atg16l1, Gnb2, Rab3a, Cask, Ash1l, Atf7ip, Tsen34, Tfg, Tgfbr1, Prlr, Ppp1r10, Lrrc1, Marcks, Crk, Arntl	20512, 94186, 12632, 54673, 73178, 12014, 100226, 77579, 64144, 77040, 14693, 19339, 12361, 192195, 54343, 66078, 21787, 21812, 19116, 52040, 214345, 17118, 12928, 11865
Calmodulin-binding	GO:0005516	2,73E-05	n/a	4	0,173120729	114	Strn3, Myh10, Cask, Marcks	94186, 77579, 12361, 17118
ADP-binding	GO:0043531	2,67E-04	n/a	2	0,024297646	16	Myh10, Cyb5r3	77579, 109754
Actin-binding	GO:0003779	8,10E-04	n/a	4	0,419134396	276	Cfl2, Wasl, Myh10, Marcks	12632, 73178, 77579, 17118
Enzyme-binding	GO:0019899	2,31E-03	n/a	4	0,557327259	367	Tgfbr1, Prlr, Ppp1r10, Marcks	21812, 19116, 52040, 17118
Transferase activity	GO:0016740	2,83E-03	n/a	8	2,472285497	1628	Sh3glb1, Cask, Ash1l, Tgfbr1, Mgat4a, Mark3, Crk, Fntb	54673, 12361, 192195, 21812, 269181, 17169, 12928, 110606
Nucleotide-binding	GO:0000166	2,85E-03	n/a	9	3,056947608	2013	Myh10, Clcn5, Cyb5r3, Rab3a, Cask, Cpeb2, Tgfbr1, Tardbp, Mark3	77579, 12728, 109754, 19339, 12361, 231207, 21812, 230908, 17169
G-quadruplex RNA-binding	GO:0002151	3,03E-03	n/a	1	0,003037206	2	Aff2	14266
tRNA-intron endonuclease activity	GO:0000213	3,03E-03	n/a	1	0,003037206	2	Tsen34	66078
Protein farnesyltransferase activity	GO:0004660	3,03E-03	n/a	1	0,003037206	2	Fntb	110606
High-affinity glutamate transmembrane transporter activity	GO:0005314	3,03E-03	n/a	1	0,003037206	2	Slc1a3	20512
Cytoskeletal protein-binding	GO:0008092	3,28E-03	n/a	4	0,615034169	405	Cfl2, Wasl, Myh10, Marcks	12632, 73178, 77579, 17118
Catalytic activity	GO:0003824	3,88E-03	n/a	15	7,357630979	4845	Sh3glb1, Inpp5a, Myh10, Gnb2, Cyb5r3, Cask, Ash1l, Atf7ip, Tsen34, Tgfbr1, Mgat4a, Mark3, Pde4b, Crk, Fntb	54673, 212111, 77579, 14693, 109754, 12361, 192195, 54343, 66078, 21812, 269181, 17169, 18578, 12928, 110606
alpha-1,3-mannosylglycoprotein 4-beta-N-acetylglucosaminyltransferase activity	GO:0008454	4,55E-03	n/a	1	0,004555809	3	Mgat4a	269181
Protein phosphatase 1 binding	GO:0008157	4,55E-03	n/a	1	0,004555809	3	Ppp1r10	52040
Protein complex binding	GO:0032403	4,96E-03	n/a	3	0,344722855	227	Strn3, Cask, Tgfbr1	94186, 12361, 21812
Cytokine binding	GO:0019955	6,77E-03	n/a	2	0,123006834	81	Tgfbr1, Prlr	21812, 19116
Poly-pyrimidine tract binding	GO:0008187	7,57E-03	n/a	1	0,007593014	5	Cpeb2	231207
Cytochrome-b5 reductase activity	GO:0004128	7,57E-03	n/a	1	0,007593014	5	Cyb5r3	109754
Farnesyltranstransferase activity	GO:0004311	7,57E-03	n/a	1	0,007593014	5	Fntb	110606
Transforming growth factor beta receptor activity, type I	GO:0005025	9,08E-03	n/a	1	0,009111617	6	Tgfbr1	21812
Neurexin binding	GO:0042043	9,08E-03	n/a	1	0,009111617	6	Cask	12361
Transforming growth factor beta binding	GO:0050431	9,08E-03	n/a	1	0,009111617	6	Tgfbr1	21812
Actin-dependent ATPase activity	GO:0030898	9,08E-03	n/a	1	0,009111617	6	Myh10	77579
Nucleic acid binding	GO:0003676	9,71E-03	n/a	9	3,690205011	2430	Aff2, Bach2, Ash1l, Cpeb2, Tsen34, Tardbp, Ehf, Ppp1r10, Arntl	14266, 12014, 192195, 231207, 66078, 230908, 13661, 52040, 11865

The combined list of unique gene targets (2548 genes, 2275 mapped to GO Terms) derived from the analysis of five miRNA target databases was analysed for enrichment of molecular function Gene Ontology terms using the Genomatix Genome Analyser GeneRanker tool (www.genomatix.de). El Dorado version 1210. GO terms selected have a probability of enrichment less than 0.01.

### The mRNA for N-WASP is targeted by miR-142-3p

**Figure 2:**
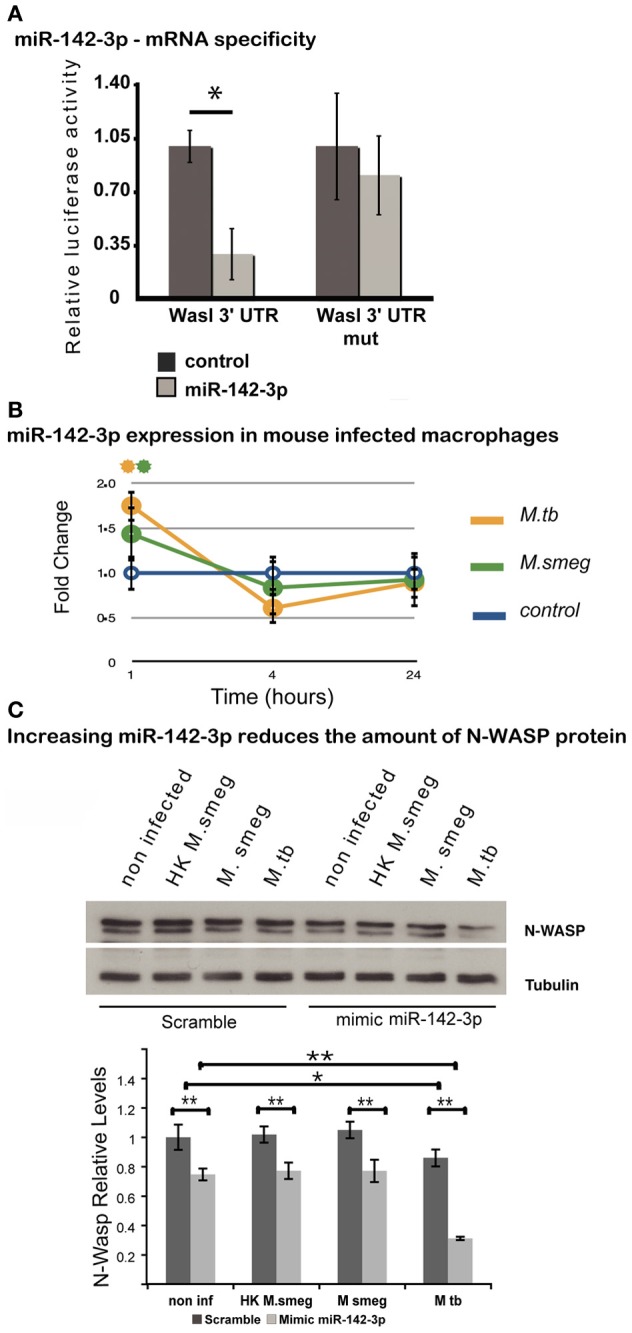
**N-Wasp is a target of miR-142-3p. (A)** Luciferase assay showing the specific targeting of the 3′UTR of the mRNA of Wasl by the miR-142-3p. Data are represented as the mean fold change per sample ± SD (^*^P ≤ 0.01). **(B)** Relative expression of miR-142-3p in J774A.1 macrophages infected with *M. smegmatis* or *M. tuberculosis* (MOI 10), as measured by the EXIQON (DK) microRNA qPCR services. Data is represented as the mean fold change per sample ± SD at 1, 4, and 24 h post-infection (^*^P ≤ 0.05 relative to control). **(C)** Relative protein levels by western blot in J774A.1 macrophages transfected with mimics of miR-142-3p or not, and that of internalized mycobacteria (MOI 10) after a 1-h challenge. N-Wasp levels are relative to that of α/β-Tubulin. A representative blot from three independent experiments is shown with the densitometry quantification: quantification of the relative levels of N-Wasp in infected macrophages, treated with either mimics of miR-142-3p or scramble (^*^P ≤ 0.01; ^**^P ≤ 0.001).

Given its involvement during early events of phagocytosis (McGee et al., 2001; Caron et al., 2006; Park and Cox, 2009; Dart et al., 2012), and the strong prediction for it being a gene target for miR-142-3p (Tables 1 and 3), we asked whether this miRNA may target the 3′-UTR mRNAs Wasl (mRNA for N-Wasp). To accomplish this, we used a dual luciferase reporter vector system in which the 3′-UTR sequence for Wasl was inserted into the pmirGLO dual-luciferase target expression vector. The system allows one to quantitatively assessing the transcript activity, upon binding to a potential miRNA target, thus validating the specificity of miRNA-mRNA 3′-UTR pair interaction. As shown in Figure 2A, the relative luciferase activity is lower in the pair miR-142-3p/Wasl relative to the control, indicating that the miR-142-3p targets the Wasl mRNA 3′-UTR with high probability (*P* < 0.01). By contrast, when a plasmid bearing a mutated form of the Wasl sequence by one nucleotide substitution was tested, there was no decrease in luciferase activity detected. Altogether, this demonstrates that Wasl is indeed a target of miR-142-3p.

### The modulation of miR-142-3p expression partially influences the amount of N-WASP protein

Our previous results confirmed that miR-142-3p targets the mRNA sequence of N-Wasp, suggesting it may influence the level of N-Wasp protein, and consequently, the phagocytosis process. In order to test this hypothesis, we first verified whether the pathogenic Mtb strain H37Rv was capable of modulating the miR-142-3p expression as *M. smegmatis*. We performed a time course of infection (1, 4, and 24 hpi) with either strains and measured the miR-142-3p expression by qPCR analysis. As shown in Figure 2B, Mtb significantly induced the expression levels for miR-142-3p at 1 hpi similar to that obtained with *M. smegmatis* challenge. Yet, similar to the pattern obtained during infection with *M. smegmatis*, the induction caused by Mtb was short-lived, as the expression levels for miR-142-3p drops considerably at 4 hpi and remains low at 24 hpi (Figure 2B). Therefore, if miR-142-3p plays a role in regulating N-Wasp activity, then it is only early on during the interaction with mycobacteria, such as the phagocytosis process.

Next, to examine whether miR-142-3p is able to regulate N-Wasp expression in Mφs, we conducted gain-of-function and loss-of-function experiments in order to measure N-Wasp protein levels during mycobacterial infection. We employed the use of either “mimics” of miR-142-3p to imitate and increase its behavior (gain-of-function), or “inhibitors” to nullify its activity (loss-of-function), as described in Materials and Methods. In this manner, Mφs were transfected with mimics or inhibitors of miR-142-3p and subsequently challenged with either *M. smegmatis* or Mtb for 1 h. Whole cell extracts were then prepared for western blot analyses. As shown in Figure 2C, our results revealed that Mtb alone can partially down-regulate the levels of N-Wasp protein (~20% less relatively to non-infected cells), as challenge with *M. smegmatis* (either alive or heat-killed) failed to alter its expression level. Likewise, the use of mimics partially decreased the N-Wasp protein (~20% less, relative to miRNA control) in non-infected cells. Strikingly, there seemed to be an additive effect with the use of mimics and challenge with Mtb since the level of N-Wasp protein was drastically reduced (~50% compared to miRNA control). This additive effect, however, was not observed during challenge with *M. smegmatis* (either alive or heat-killed) (Figure 2C). Unlike the treatment with mimics, Mφs transfected with inhibitors of miR-142-3p and subsequently challenged with these mycobacterial strains showed no significant change in the protein levels of N-Wasp, implying that a compensatory effect was occuring (Data not shown).

### miR-142-3p activity correlates with a reduction of the amount of internalized mycobacteria per Mφ

**Figure 3:**
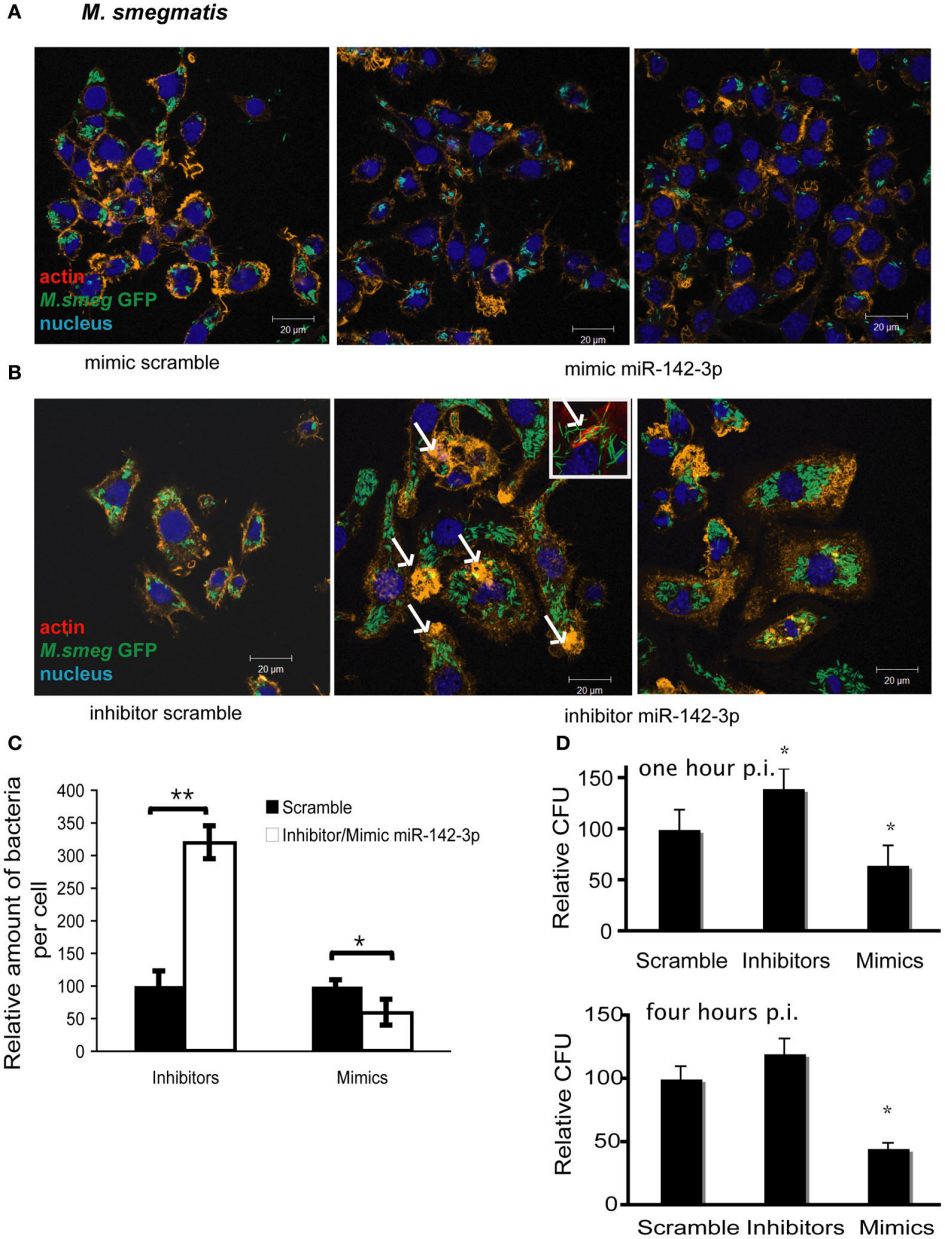
**miR-142-3p activity correlates with a reduction of the amount of internalized *M. smegmatis* per Mφ**. Confocal microscopy showing quantitative and qualitative analysis of J774A.1 macrophages treated with miR-142-3p mimics **(A)** or miR-142-3p inhibitors **(B)**, and challenged with *M. smegmatis* (MOI 10) for 4 h. Arrows indicate phagocytic cups. Blue (DAPI), green (*M. smegmatis* GFP), and light red/orange (Rhodamine-Phalloidin). Bar: 20 μm. **(C)** Quantification of the relative amount of bacteria per macrophage treated with mimics or inhibitors of miR-142-3p. Data is represented as the mean area of bacteria per macrophage, per sample ± SEM at 4 h post infection (^*^P ≤ 0.05; ^**^P ≤ 0.01). Data was analysed using ImageJ macros (http://www.formatex.info/microscopy4/614-621.pdf). **(D)** Colony forming units assay (CFU) of *M. smegmatis*-infected macrophages (MOI 0.1) either for 1 (top) or 4 (bottom) h, and under the treatment with mimics or inhibitors of miR-142-3p (^*^P ≤ 0.05).

**Figure 4:**
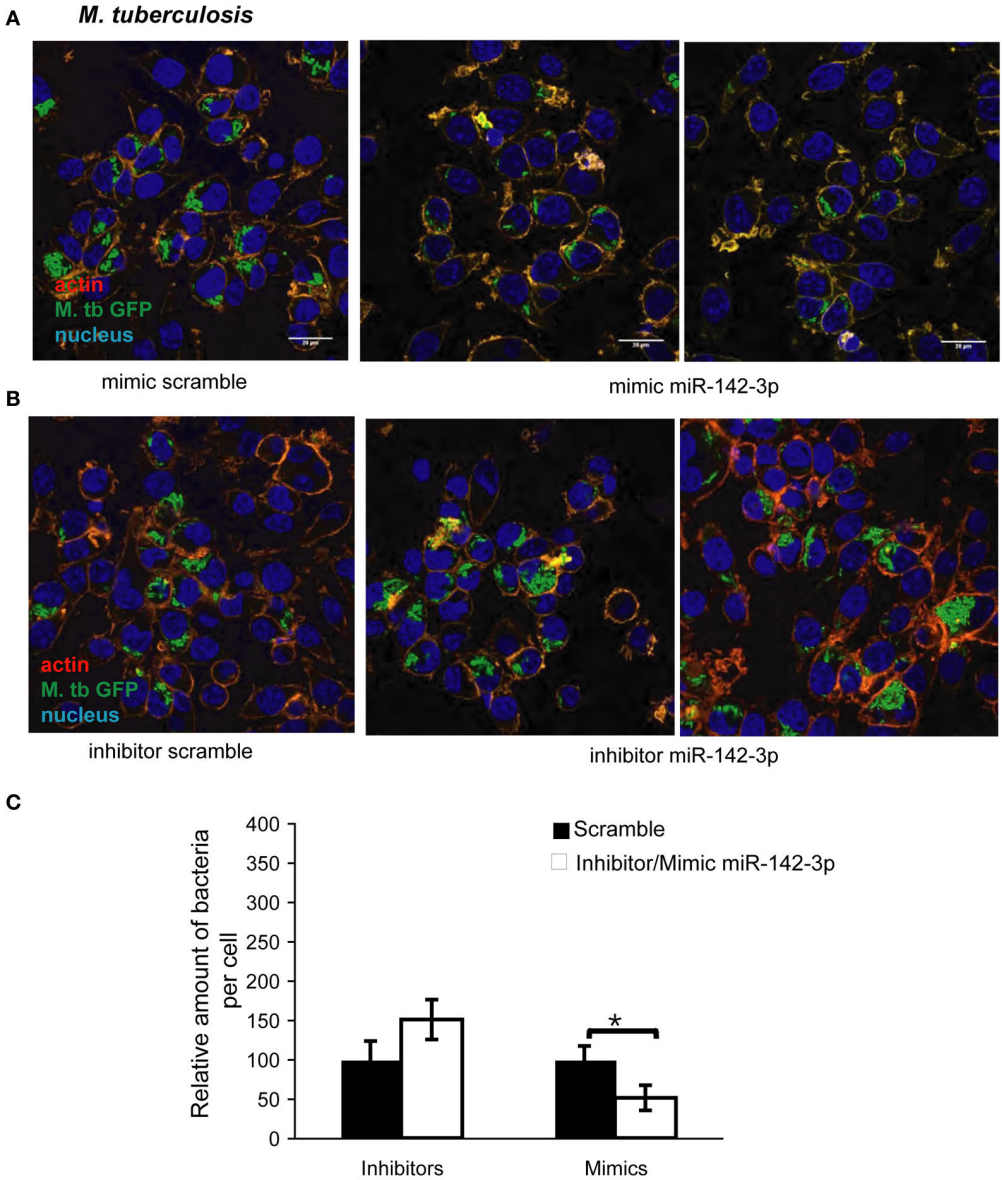
**miR-142-3p activity correlates with a reduction of the amount of internalized *M. tuberculosis* per Mφ**. Confocal microscopy showing quantitative and qualitative analysis of J774A.1 macrophages treated with miR-142-3p mimics **(A)** or miR-142-3p inhibitors **(B)**, and challenged with *M. tuberculosis* (MOI 10) for 4 h. Blue (DAPI), green (H37Rv-eGFP), and Light red/orange (Rhodamine-Phalloidin). Bar: 20 μm. **(C)** Quantification of the relative amount of bacteria per macrophage treated with mimics or inhibitors of miR-142-3p. Data is represented as the mean area of bacteria per macrophage, per sample ± SEM at 4 h post infection (^*^P ≤ 0.05). Data was analysed using ImageJ macros (http://www.formatex.info/microscopy4/614-621.pdf).

The reduction of N-Wasp expression by the treatment mimicking miR-142-3p of Mφs infected with either mycobacterial strain, prompted us to investigate for a possible role for this miRNA in controlling the early stages of phagocytosis. To assess this, we again employed the use of either “mimics” or “inhibitors” of miR-142-3p activity. Our hypothesis predicted a decrease in bacterial intake with the use of the mimics and an increase in the presence of inhibitors. As depicted in Figure 3A, confocal analysis confirmed that Mφs treated with the mimics resulted in a reduced amount of intracellular *M. smegmatis* at 4 hpi when compared to the negative control (scrambled miRNA). By contrast, Mφs treated with the inhibitors led to a dramatic increase in the amount of intracellular *M. smegmatis*. This effect was accompanied by distinct morphological changes in terms of cell size and large numbers of phagocytic cups, as compared to Mφs treated with the mimics (Figure 3B). The quantification analysis of the confocal analysis is illustrated in Figure 3C. Furthermore, the bacterial intake under these conditions was also measured at 1 or 4 hpi by CFU assays; this alternative quantitative method revealed similar effects to those obtained by confocal microscopy (Figure 3D). In the case for Mtb, Mφs treated with the miR-142-3p mimics resulted in a significant reduced amount of intracellular Mtb when compared to the miRNA control at 4 hpi, thus confirming our previous results with *M. smegmatis* challenge (Figure 4A). However, we were surprised that treatment of Mφs with the inhibitors did not lead to a significant increased level of intracellular Mtb, as it was the case for *M. smegmatis* (Figure 4B). The quantification analysis of the confocal analysis is provided in Figure 4C.

### Mtb induces miR-142-3p expression while decreasing that of N-WASP in human primary Mφs

**Figure 5:**
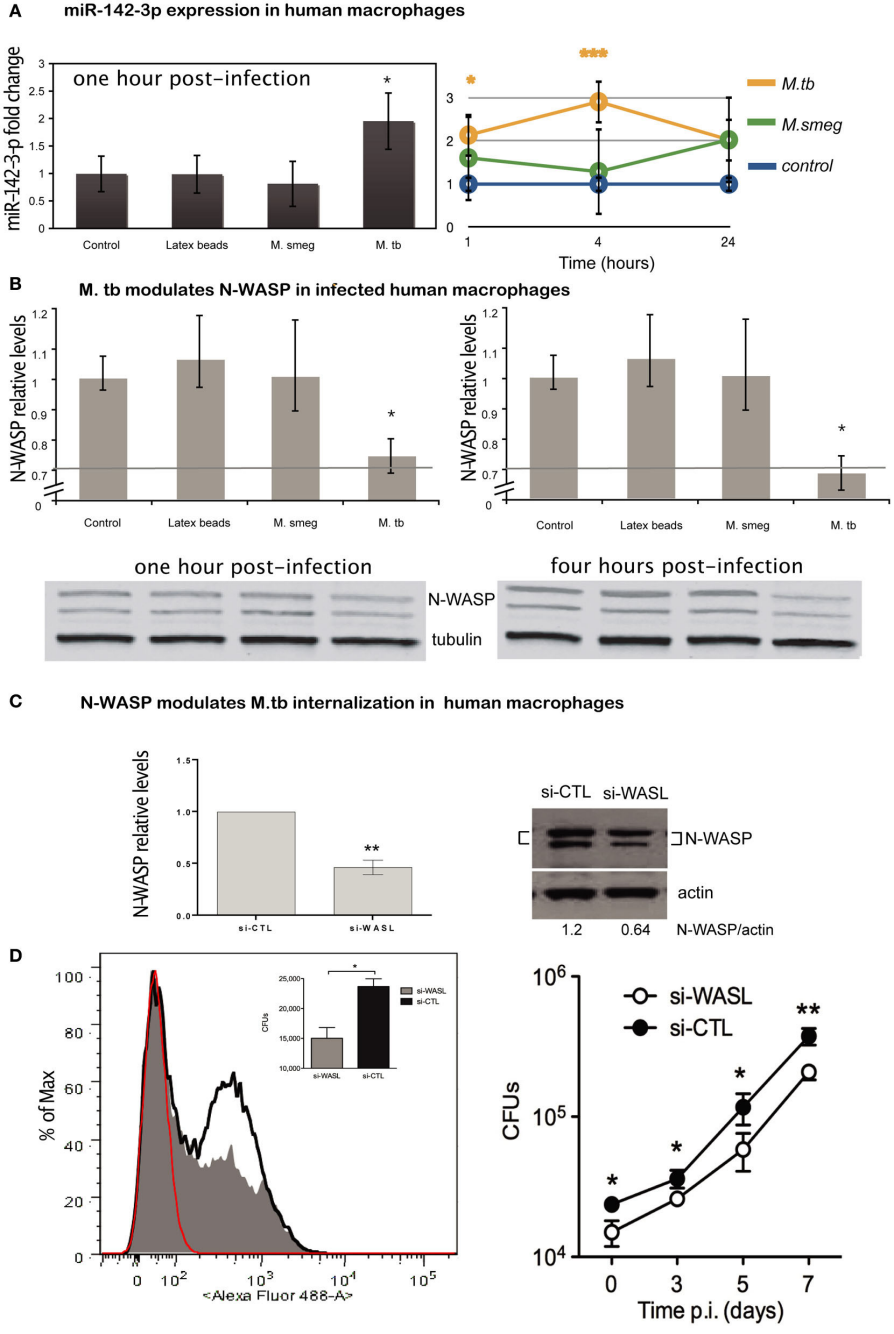
**Expression of miR-142-3p and N-Wasp levels in infected human primary macrophages. (A)** Left: relative expression of miR-142-3p under *M. smegmatis*, *M. tuberculosis* or latex beads, exposure (1 h) of macrophages, as measured by qPCR analysis. Right: relative expression of miR-142-3p in human macrophages infected with *M. smegmatis* or *M. tuberculosis* as the indicated time points, as measured by EXIQON (DK) microRNA qPCR services. **(B)** Relative protein levels by western blot analysis. N-Wasp levels relative to α/β-Tubulin either at 1 h post-infection with 20% reduction (upper left) or 4 h post-infection with 40% reduction (upper right). A representative blot (bottom) from three independent experiments is shown with densitometry quantification for each time point; data are represented as the mean fold change per sample ± SD (^*^P = 0.05). **(C)** The siRNA-mediated inactivation of N-Wasp (WASL) was performed as described in materials and method. Transfection of the siRNA SMARTpool targeting N-Wasp (si-WASL) resulted in an average of about 54% reduction of the protein level relative to that of the transfection with a non-targeting control siRNA pool (si-CTL). A representative western blot analysis (right) illustrates the gene inactivation obtained from four independent experiments (left); data are represented as the mean fold change relative to control sample (set arbitrarily at 1) ± SD (^**^P ≤ 0.01). **(D)** Left: phagocytosis of H37Rv-eGFP by human macrophages either inactivated for N-Wasp (si-WASL, gray) or transfected with the non-targeting siRNA pool (si-CLT, black), was analysed either by flow cytometry (histogram) analysis at MOI 10, or by CFU (inlet) assay at MOI 0.1, after 4 h of infection. Red indicates the fluorescence background of non-infected macrophages. The median fluorescence intensities (MFI) are as follow: 36 (non-infected), 117 (si-WASL) and 189 (si-CTL). Right: H37Rv-eGFP proliferation as measured by CFU analysis for different time points (days) for the same cellular conditions and donor as described for left panel. The data are representative of two independent experiments done in triplicates ± SD (^*^P = 0.05; ^**^P = 0.01).

MiR-142-3p is highly conserved across species (Kozomara and Griffiths-Jones, 2011). Our findings in the murine model suggest that Mtb induces the timely expression of miR-142-3p in order to down-modulate the function of N-Wasp protein, and therefore, modulate the uptake by phagocytic cells. Given the Mtb is the etiological agent of TB in humans, and that human Mφs are the primary replication site for this obligate intracellular pathogen, we used primary human monocyte-derived Mφs in order to investigate whether the expression of miR-142-3p leads to a subsequent reduction of N-Wasp and alter the rates of phagocytosis. Human Mφs were exposed to latex beads (to induce a “sterile” phagocytosis process), or challenge with either *M. smegmatis* or Mtb, and the miR-142-3p expression was quantified by qPCR analysis. Our results indicate that while Mtb infection of human Mφs specifically induces the expression of miR-142-3p, challenge with *M. smegmatis* or exposure to latex beads failed to do so (Figure 5A, left).

Next, we measured whether the difference in miR-142-3p expression obtained from the challenge with either mycobacterial strain at 1 hpi is sustained throughout infection. Indeed, we observed that it became more pronounced at 4 hpi, with an eventual decline at 24 hpi, but remaining always above the expression level of non-infected cells (Figure 5A, right). Coincidently, the protein levels of N-Wasp were slightly, but significantly reduced (~20%) at both 1 and 4 h upon Mtb infection, while remaining unaffected by the challenge with *M. smegmatis* or exposure to latex beads (Figure 5B). These results, along with those obtained in the mouse model context, prompted us to examine whether the decrease in N-Wasp expression had any functional consequence for the phagocytosis process of Mtb. To accomplish this, we used a siRNA-based protocol that we have adapted and improved to effectively inactivate the gene expression in primary human Mφs (Lefèvre et al., 2013). Using this protocol, we obtained a significant reduction (on average ~54%) of N-Wasp protein levels in human Mφs (Figure 5C). This partial inactivation of N-Wasp protein resulted in a decrease of Mtb intake as measured either by flow cytometry (Figure 5D, left), or by CFU assays at 4 hpi (Figure 5D, left inlet); this pattern continued over a time course of 1 week (Figure 5D, right). Altogether, these results suggest that the modulation of N-Wasp function via miR-142-3p might contribute to the phagocytosis process of Mtb in human cells.

## Discussion

TB is still one of the major causes of death due to a single infectious agent (Mtb) with 1.7 million cases in 2009. There is an urgent need for scientific research that may improve treatment, diagnoses and prevention of TB. A better understanding of how Mtb subverts host cells and hijacks cellular mechanisms is necessary. A case in point is the inhibition by Mtb of phagolysosome biogenesis in Mφs, in which the regulation of actin-mediated events plays a central role. For these reasons, we decided to investigate the role of miRNAs as a potential novel mechanism in the regulation of actin-mediated events influencing the process of phagocytosis within the context of mycobacteria infection. Taken all our observations together, we believe this study makes three significant contributions to this emerging field.

The first major contribution is our description of miRNA modulation upon mycobacterial challenge. To our knowledge, this is the first study to undertake the assessment of miRNA expression patterns during the early stages of the mycobacteria-Mφ interaction. Our global transcriptomic approach revealed that 36 miRNAs are significantly modulated, of which 27 up-regulated and 9 down-regulated. Beyond the identification of miR-142-3p as a key candidate, and its implications (discussed below), there are 9 miRNAs (i.e., miR: 29, 93, 101, 181, 207, 329, 451, 574, and 684) in our list that are reported to be similarly modulated in multiple mycobacterial infection contexts (Fu et al., 2011; Ma et al., 2011; Sharbati et al., 2011; Wang et al., 2011; Yi et al., 2012). Of special interest is the miR29 family, since its members are known to play a major role in human diseases (Wang et al., 2008; Park et al., 2009; Xiong et al., 2010). Not only are there different reports about the up-regulation of miR-29a in patients with active TB (Fu et al., 2011; Yi et al., 2012), but also its role in the innate and adaptive immune responses to mycobacterial infection has been recently described (Ma et al., 2011; Sharbati et al., 2011). Indeed, miR-29 inhibits the production of IFNγ, a crucial cytokine for the microbiocidal response against intracellular pathogens. The fact that Mtb upregulates miR-29 expression during the course of the infection suggests that it also modulates IFNγ production to tilt the immune response in its favor (Ma et al., 2011). Therefore, the case of miR-29 best illustrates the potential of using microRNA modulation as microbial strategy to circumvent the immune system (Eulalio et al., 2012b), and it validates the exclusive list of miRNAs obtained in this study.

The second major contribution of this study is identification of miR-142-3p as a key candidate involved in the regulation of actin dynamics required in phagocytosis. The induction of miR-142-3p as detected by our microarray analysis was confirmed by qPCR analysis with both non-virulent and virulent mycobacterial strains. In the murine Mφs, the challenge with these mycobacteria resulted in a similar short-lived up-regulation of this miRNA, but only during the first hour in infection. In primary human Mφs, however, only the challenge with virulent Mtb resulted in rapid high levels of miR-142-3p over background, peaking at 4 h and declining thereafter. The discrepancy between the results from different between species can be explained by the fact that Mtb has co-evolved as with humans with predilection for Mφs as primary reservoirs, or by the well-known differences between cell lines and primary cells (e.g., pathogen recognition receptor repertoire), among other reasons.

The expression of miR-142-3p was first reported to be exclusive to cells of the hematopoietic system, with aberrant dys-regulation in T-cell and B-cell leukemia (Bellon et al., 2009). In addition, this miRNA was observed during normal granulocytopoiesis (Careccia et al., 2009), and more recently, characterized as a key regulator (along with miR-29) for normal myeloid leukocyte differentiation (Wang et al., 2012). In fact, the down-regulation of these two miRNAs is associated with acute myeloid leukemia development (Wang et al., 2012). *Bona fide* targets for miR-142-3p include RAC1, CD133, IL-6, and ADCY9 (Huang et al., 2009; Bissels et al., 2011; Sun et al., 2011; Wu et al., 2011). Of particular interest, the latter two gene targets are of relevance to our study. In the case of IL-6, the high expression of miR-142-3p in murine and human dendritic cells was demonstrated to be essential to regulate the biosynthesis of this cytokine and prevent endotoxin-induced mortality (Sun et al., 2011). In the case for ADCY9, miR-142-3p was reported to target this gene, resulting in the regulation of cAMP that is crucial to the suppressive effect enforced by in CD4(+) CD25(−) regulatory T cells during the resolution of the inflammatory response (Huang et al., 2009). Indeed, the control of these two gene targets indicates miR-142-3p can influence both arms of the immune system, and that aberrant expression of this miRNA (e.g., due to a microbial hijacking strategy), could then result in a defective inflammation response that is counter-productive to host fitness. Finally, while miR-142-5p is generated along with miR-142-3p after maturation of the pre-miR-142 (Wu et al., 2009), it ultimately failed to target the mRNA sequence for Cdc42ep4 mRNA 3′-UTR, a Rho GTPase that regulates signaling pathways controlling diverse cellular functions including endocytosis (Data not shown). As Cdc42ep4 was the only potential predicted target (by miRDB and microrna.org) with the potential to regulate ABP activity, we therefore ruled out that miR-142-5p can influence the early events of phagocytosis in concert with miR-142-3p.

The third contribution of this study is the suggestion that a novel but general strategy in the context of mycobacteria infection is the role of miRNAs in modulating mycobacterial uptake by phagocytic cells, revealed by the partial and temporal inhibition of N-Wasp activity via miR-142-3p. Collectively, we demonstrate that: (1) mycobacteria infection of Mφs results in a short-lived induction of miR-142-3p, and in the case of Mtb, accompanied by a partial decrease of N-Wasp protein levels (2) N-Wasp mRNA (Wasl) is a direct target for miR-142-3p, (3) miR-142-3p leads to a significant decrease of intracellular mycobacteria intake by Mφs, and (4) the siRNA-mediated inactivation of N-Wasp in human Mφs affects the initial rate of phagocytosis of Mtb. Furthermore, an analysis of the 3′-UTR sequence of the N-Wasp mRNA revealed that additional miRNAs might bind to this target. Based on the microRNA.org tool (Betel et al., 2008), we identified miR-377, miR-32, miR-410, miR-19b, and let-7f, as potential candidates to bind to the 3′-UTR sequence of the N-Wasp mRNA, ultimately suggesting that a group of different miRNAs might be directly controlling the expression of a single target in a concerted manner.

The regulation of N-Wasp activity is important, since this protein is known to be involved in actin dynamics required during the invasion of host cells by several pathogens. For instance, salmonella induces actin assembly via N-Wasp and therefore bacteria uptake by non-professional phagocytes through a type III secretion system (Unsworth et al., 2004). Additional examples include the actin filament assembly and regulation associated with the establishment of actin pedestals during enteropathogenic *E. coli* EPEC (Kalman et al., 1999) and Vaccinia virus invasion (Frischknecht et al., 1999), and the actin tail-propulsion based invasion of host cells by shigella (Suzuki et al., 1998) and listeria (Gouin et al., 1999). More relevant for our study, actin tail based movement dependent on N-Wasp during mycobacteria infection was also described for *Mycobacterium marinum*, a close relative of Mtb (Stamm et al., 2003, 2005). Finally, a knock-out of N-Wasp was associated with a defective immune response to *M. bovis* BCG (Andreansky et al., 2005). All things considered, our findings strongly suggest that effective modulation of N-Wasp activity via miR-142-3p can influence the rate of bacterial intake by Mφs, and to our knowledge, this is the first description of a microbial strategy employing the use of miRNAs to regulate actin-mediated events leading to phagolysosome biogenesis. The central role of N-Wasp in this process is indeed supported by the case of *Yersinia pseudotuberculosis*, which modulates the activity of N-Wasp to control its internalization in host cells (McGee et al., 2001).

As mentioned, one of the best strategies for an invading microbe is to manipulate the early steps of the interaction with Mφs in order to avoid the activation of the microbicidal mechanisms, as best illustrated by the ability of Mtb to inhibit phagolysosome biogenesis in Mφs (Deretic et al., 2004). A recurring theme among the most successful intracellular microbes is the targeting of the host cell's cytoskeleton, implying that it favors pathogenic entry into favored cells, movement within and between target cells, influence in vacuole formation and remodeling, and control of phagocytosis. We argue that the miRNA list provided in this report have potential roles in these activities, all in some way relying on the host cell cytoskeleton, as evidenced by the role of miR-142-3p in partially controlling N-Wasp in activity in the process of phagocytosis. All in all, this study promotes the concept of miRNA modulation as a new venue for Mtb to shift the Mφ response in its favor, adding yet another chapter in the arms race of host-microbe coevolution.

## Author contributions

Conceived and designed the experiments: Paulo Bettencourt, Elsa Anes, Geanncarlo Lugo-Villarino. Performed the experiments: Paulo Bettencourt, Sabrina Marion, Leonor F. Santos, David Pires, Nuno Carmo, Jonathon Blake, Vladimir Benes, Geanncarlo Lugo-Villarino and Claire Lastrucci. Analyzed the data: Paulo Bettencourt, Jonathon Blake, Gareth Griffiths, Geanncarlo Lugo-Villarino, Claire Lastrucci, Olivier Neyrolles and Elsa Anes. Wrote the paper: Paulo Bettencourt, Geanncarlo Lugo-Villarino and Elsa Anes.

### Conflict of interest statement

The authors declare that the research was conducted in the absence of any commercial or financial relationships that could be construed as a potential conflict of interest.
